# Age differences in the association of body mass index-defined obesity with abdominal aortic calcification

**DOI:** 10.3389/fendo.2024.1336053

**Published:** 2024-03-13

**Authors:** Tangmeng Guo, Lili Huang, Zhijian Luo, Huabo Zheng, Shengshuai Shan, Bei Cheng

**Affiliations:** ^1^ Department of Geriatrics, Union Hospital, Tongji Medical College, Huazhong University of Science and Technology, Wuhan, China; ^2^ Institute of Gerontology, Union Hospital, Tongji Medical College, Huazhong University of Science and Technology, Wuhan, China; ^3^ Department of Clinical Laboratory Medicine, People’s Hospital of Dongxihu District Wuhan City and Union Dongxihu Hospital, Huazhong University of Science and Technology, Wuhan, China; ^4^ Department of Neurology, The First Hospital of Jingzhou, Clinical Medical College, Yangtze University, Jingzhou, China; ^5^ Departemnt of Cardiology, Pneumology, Angiology and Internal Medicine Intensive Care, University Hospital, Rheinisch-Westfälische Technische Hochschule Aachen, Aachen, Germany; ^6^ Clinical and Experimental Therapeutics Program, Department of Clinical and Administrative Pharmacy, University of Georgia, Augusta, GA, United States

**Keywords:** age, obesity, abdominal vascular calcification, NHANES, body mass index

## Abstract

**Objectives:**

In cardiovascular disease, previous studies have suggested young age as one of the reasons to explain the obesity paradox. This study attempts to provide a different opinion on this claim through unexpected findings.

**Methods:**

We used a cross-sectional analysis of the US nationally representative data, total of 10,175 participants were recruited in 2013-2014 from NHANES. A total of 947 participants were selected to be included in this study through inclusion criteria and exclusion criteria for statistical analysis of the relationship between obesity and abdominal aortic calcification(AAC). Smooth curve fitting and multivariate regression analyses were conducted to examine the associations of obesity with AAC after adjusting for age, gender and associated variates.

**Results:**

Depending on the age of the population, the relationship between obesity and AAC showed the different outcome. Obesity was associated with the lower risk of AAC among individuals older than 52 years of age. According to the difference of adjusted covariates, the AAC scores in the obesity group decreased by 0.92, 0.87, and 1.11 for 52 years old or older individuals. In particular, the risk of AAC was lower for patients with obesity with the following characteristics: male, low LDL, low triglyceride, DM, non-cancer patient, smoking, drinking, vigorous work activity, low annual household income, education of 9 – 11th grades and non-Hispanic white.

**Conclusions:**

In US, adults aged 52 years or older, obesity was associated with decreased AAC risk. Older age may be one potential reason for the obesity paradox.

## Introduction

Cardiovascular disease (CVD) remains the leading cause of death globally (1). Abdominal aortic calcification (AAC) is significantly associated with CVD, and the circularity of the calcification independently adds to the cardiovascular risk (2–4). Previous studies have found that AAC results in increased aortic stiffness, isolated systolic hypertension, decreased organ perfusion, left ventricular hypertrophy, diastolic dysfunction, and heart failure with preserved ejection fraction (5–8). In addition, AAC can measure advanced atherosclerosis, which predicts CVD morbidity and mortality independently of traditional CVD risk factors (9).

A recent study found that obesity accelerated vascular calcification (VC) *in vivo*, which plays an important role in VC response to cholecalciferol *in vivo*, resulting in increased ectopic mineralization signaled by specific osteochondrogenic program activation and associated positive, hypotrophic vascular remodeling (10). Various measures of obesity were associated with increased progression of coronary artery calcification (CAC) (11–14). However, over the past 25 years, quite a few studies have demonstrated a strong “obesity paradox.” This paradox suggests that although obesity has a detrimental effect on risk factors associated with cardiovascular disease and many other chronic diseases, patients with cardiovascular disease and who are overweight or obese tend to have a better prognosis than thinner patients (15). One study has suggested young age as one of the reasons to explain the obesity paradox (16). Therefore, it is necessary to study the association between obesity and AAC by age stratification.

To fill these knowledge gaps, it is necessary to reveal the relationship between obesity and AAC with data based on population epidemiology. This study analyzed the association of obesity and AAC from National Health and Nutrition Examination Survey (NHANES) in a nationally representative sample of U.S. adults. The study’s strengths include its large, representative national sample and its consistent use of standardized methods.

## Methods

### Study participants

**Figure 1:**
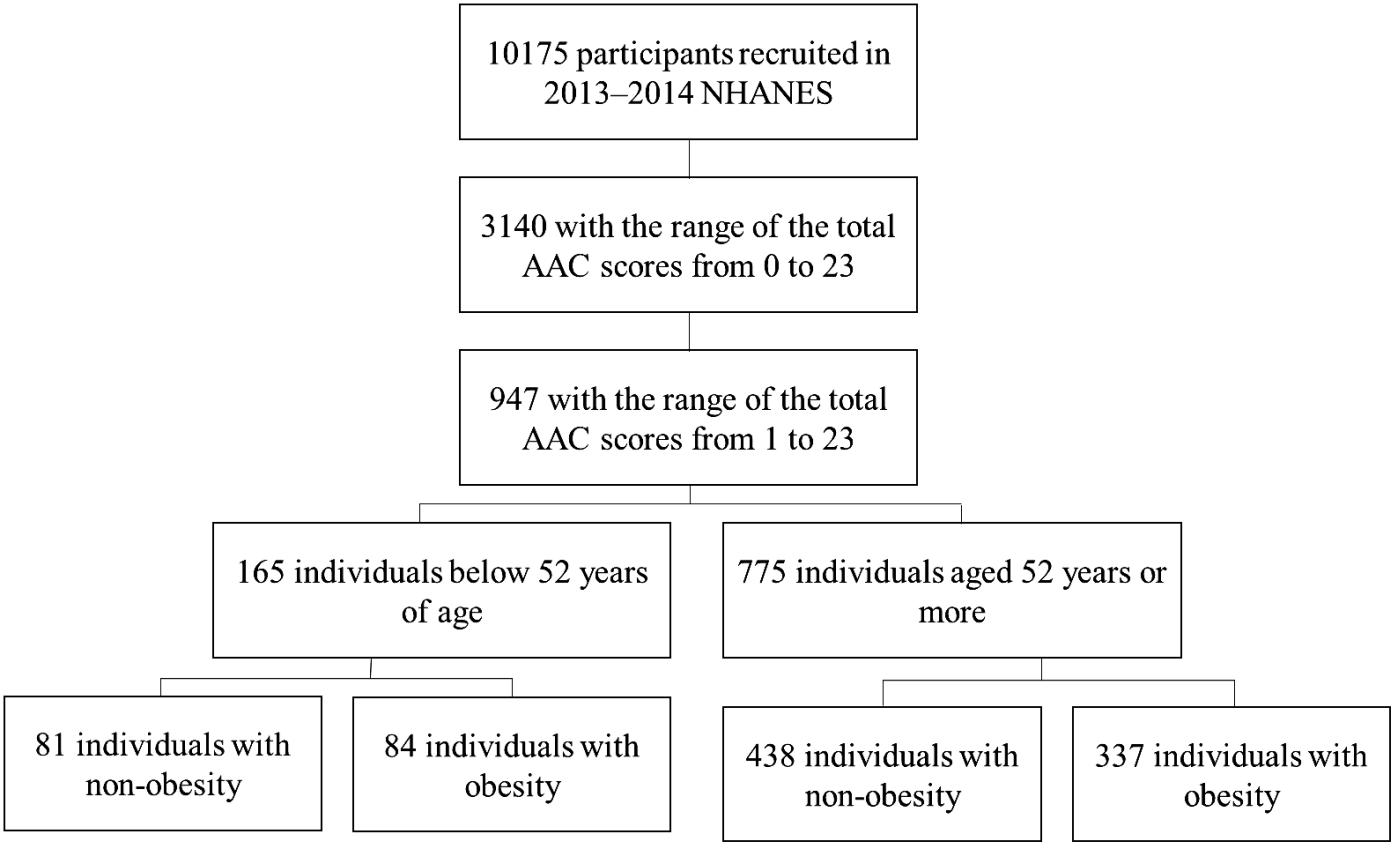
Flow-chart of study participants.

NHANES was a stratified, multistage probability sampling method to select a series of cross-sectional, nationally representative samples. It was designed to assess the health and nutritional status of the US general population (17). The current analyses were limited to participants aged 40 years or older who completed the lateral spine scan of instant vertebral assessment and whose L1-L4 vertebrae are valid in 2013–2014. The people who had one or more invalid L1-L4 vertebrae were excluded. After exclusion, 947 participants with AAC scores of 1 or more were included in the final sample for analysis. The AAC total scores were used to assess the severity of AAC. The institutional review board approved the National Center for Health Statistics study protocols. No informed consent was required because the data were anonymized. **Figure 1** depicts the flow chart of the participants’ selection process in the studies.

### Data collection

Participants completed in-home interviews and visited a mobile examination center where they underwent a physical examination and blood sample collection. A standardized questionnaire was used to collect information on age, gender, smoking history, drinking, hypertension, diabetes mellitus (DM), renal dysfunction, cancer, vigorous work activity, annual household income, education, and race/ethnicity. According to the standard questionnaire, participants were asked whether they had received a diagnosis of DM, hypertension, renal dysfunction or cancer. Race/ethnicity was categorized into Mexican American, other Hispanic, non-Hispanic white, non-Hispanic black, and other races, including “multi-racial.” Caregiver education was categorized as less than grade 9, grades 9–11 (including grade 12 with no diploma), high school graduate/general equivalency diploma or equivalent, some college, and college graduate or above. Smoking history was defined as answering “yes” to the question, “Have you smoked at least 100 cigarettes in your entire life?” Drinking history was defined as answering “yes” to the question, “Have you drunk at least 12 alcohol drinks in one year?”

During the physical examination, body mass index (BMI) was calculated as weight in kilograms divided by height in meters squared according to the measured weight and height. Obesity was defined as BMI ≥ 28.0 kg/m^2^, which was cited from the working group on obesity (18). An examination of AAC with dual-energy X-ray absorptiometry (DXA) was performed. In the scoring method for AAC, the anterior and posterior aortic walls were divided into four segments, corresponding to the areas in front of the lumbar vertebrae L1-L4. Within these eight segments, aortic calcification was recognized visually as either a diffused white stippling of the aorta extending out to the anterior and posterior aortic walls or as white linear calcification of the anterior and posterior walls. In addition, aortic calcification was scored as “0” if there was no calcification; “1” if one-third or less of the aortic wall in that segment was calcified; “2” if more than one-third but less than two-thirds was calcified; or “3” if more than two-thirds was calcified. The scores were obtained separately for the anterior and posterior aortic walls, ranging from “0” to “24” for the total score (19).

Blood pressure was measured using a mercury sphygmomanometer after the participant rested quietly in a seated position for at least 5 min by trained staff. Blood samples were collected and sent to central laboratories to determine LDL-cholesterol, triglyceride and 25-hydroxyvitamin D3 (25OHD3) using standard methods.

### Statistical analysis

The survey examination weights were used for analysis to obtain nationally representative estimates following National Center for Health Statistics guidelines (20).

All statistical tests were two-sided, and P <0.05 was considered statistically significant. Mean +/− SD for continuous variables. P-value was calculated by a weighted linear regression model. Percentages were used for categorical data. The weighted chi-squared test calculated the P-value. Smooth curve fitting was used to examine the associations of age with AAC scores when the individuals were divided into two group as obesity and non-obesity. Multivariate regression analyses were conducted to examine the associations of obesity with AAC after adjusting for age, gender, 25OHD3, LDL-cholesterol, triglyceride, SBP, DBP, smoking history, drinking, hypertension, DM, renal dysfunction, cancer, vigorous work activity, annual household income, education, and race/ethnicity. Data on LDL-cholesterol and triglyceride level were missing in 49.9% and 49.4%, respectively. We used multivariate multiple imputation analysis to impute missing values (21). Otherwise, less than 0.1% of values were missing. If ≤10% of data for the main outcome variable were missing for eligible examinees, it is usually acceptable to continue the analysis without further evaluation or adjustment as a general rule (22). All analyses were conducted using Empower (R) (www.empowerstats.com, X&Y Solutions, Inc., Boston MA) and R (http://www.R-project.org).

## Results

We defined obesity as BMI ≥ 28.0 kg/m^2^ and non-obesity as BMI<28 kg/m^2^ according to the working group on obesity in China (18). In **Table 1**, all participants were assigned into two groups, namely, obesity and non-obesity, based on their BMI values. The participants in the obesity group showed lower ages, calcification scores and 25OHD3 levels, but higher triglyceride levels and ratio of female, Mexican American, other Hispanic, non-Hispanic white, non-Hispanic black, hypertension, DM, education background of 9–11th grades, high school graduate and some college or AA degree (P<0.05). The smoking history, drinking history, vigorous work activity, annual household income and diseases of renal dysfunction and cancer were not significantly different (P>0.05).

**Table 1:** Characteristics of abdominal aortic calcification in individuals with obesity vs. non-obesity.

	Non-obesity	Obesity	P-value
**Age, years**	66.01 ± 11.94	63.22 ± 11.59	<0.01
**Gender**			0.02
** Male**	53.6	45.6	
** Female**	46.4	54.4	
**AAC scores**	5.83 ± 4.73	4.91 ± 4.10	<0.01
**25OHD3, nmol/L**	67.53 ± 26.20	63.79 ± 25.59	0.03
**LDL-cholesterol, mmol/L**	2.79 ± 1.00	2.87 ± 0.93	0.20
**Triglyceride, mmol/L**	1.44 ± 0.86	1.66 ± 0.95	<0.01
**SBP, mmHg**	132.22 ± 21.18	130.56 ± 17.92	0.21
**DBP, mmHg**	65.59 ± 17.10	68.62 ± 13.85	<0.01
**Hypertension**			0.01
** Yes**	56.8	65.3	
** No**	43.2	34.7	
**DM**			<0.01
** Yes**	20.4	32.5	
** No**	79.6	67.5	
**Renal dysfunction**			0.07
** Yes**	4.8	7.6	
** No**	95.2	92.4	
**Cancer**			0.31
** Yes**	20.4	17.8	
** No**	79.6	82.2	
**Smoking (at least 100 cigarettes in life)**			0.32
** Yes**	55.5	52.3	
** No**	44.5	47.7	
**Drinking (at least 12 alcohol drinks/1 year)**			0.12
** Yes**	73.9	69.2	
** No**	26.1	30.8	
**Vigorous work activity**			0.95
** Yes**	13.9	14	
** No**	86.1	86	
**Annual household income**			0.28
** Low**	41.9	46.5	
** Medium**	39.3	38	
** High**	18.8	15.4	
**Education**			0.01
** Less than 9th grade**	9.6	9	
** 9–11th** grades	13.7	14.3	
** High school graduate**	23.1	26.7	
** Some college or AA degree**	25	31.7	
** College graduate or above**	28.5	18.3	
**Race/ethnicity**			<0.01
** Mexican American**	7.3	13.5	
** Other Hispanic**	6.9	8.8	
** Non-Hispanic white**	52.4	54.4	
** Non-Hispanic black**	15.6	17.6	
** Other Race - Including** ** Multi-Racial**	17.7	5.7	

AAC, abdominal aortic calcification; SBP, systolic blood pressure; DBP, diastolic blood pressure; 25OHD3, 25-hydroxyvitamin D3; DM, diabetes mellitus. Mean +/− SD for: age, AAC scores, 25OHD3, LDL-cholesterol, triglyceride, SBP and DBP. Percentage for: gender, smoking history, drinking, hypertension, DM, renal dysfunction, cancer, vigorous work activity, annual household income, education and race/ethnicity.

**Figure 2:**
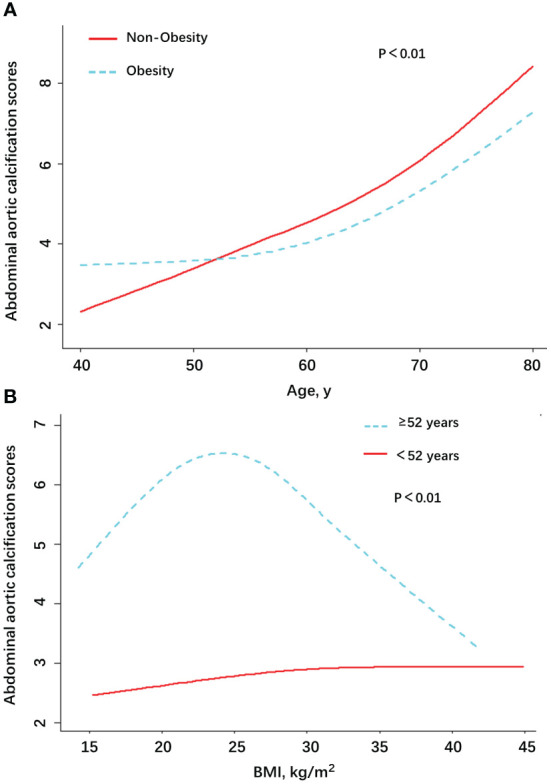
Association between AAC scores and age/BMI among patients with AAC in NHANES, by Obesity **(A)** and age **(B)**.

We used smooth curve fitting to examine the association of AAC scores and age in the individuals who were divided into two groups: obesity and non-obesity (**Figure 2A**). It could be clearly seen that, with the increase of age, the AAC scores of the non-obesity group increased gradually. The obese group showed similar results after reaching the age of 52 or older. The values of AAC scores between obesity and non-obesity group were reversed when the age was around 52 years old. The obesity group maintained higher AAC scores before 52 years old, but it was overtaken by the non-obesity group for individuals older than 52 years of age. In the **Figure 2B**, we can see that for people aged 52 and older, a BMI of about 24 is a turning point. When BMI was greater than 24, the AAC score showed a decreasing trend. The results support the obesity paradox.

Generalized additive models were used to visually assess functional relationships between the age/BMI and the risk of AAC (**Figure 2**). The stratified AAC scores by obesity or non-obesity were presented in the **Figure 2A**. The stratified AAC scores by age were presented in the **Figure 2B**. Adjusted for age, gender, 25OHD3, LDL-cholesterol, triglyceride, SBP, DBP, smoking history, drinking, hypertension, DM, renal dysfunction, cancer, vigorous work activity, annual household income, education, and race/ethnicity. Abbreviations: AAC, abdominal aortic calcification; SBP, systolic blood pressure; DBP, diastolic blood pressure; 25OHD3, 25-hydroxyvitamin D3; DM, diabetes mellitus.

Next, we used multivariate regression analyses to identify the association between obesity and AAC risk. The analysis revealed that obesity was associated with AAC scores. In people under 52 years of age, the risk of AAC appears to be higher in obesity people than in non-obesity people, although the association did not reach statistical significance (P= 0.09). When the individuals were 52 years or older, obesity was significantly negatively associated with risk of AAC (P ≤ 0.01) (**Table 2**). According to the difference of adjusted covariates, the AAC scores of the obesity group decreased by 0.92, 0.87, and 1.11, compared with those of the non-obesity group of older individuals. **Table 2** showed that the maximum effect value of obesity on AAC scores was 1.11 in the older group after adjusting for age, gender, 25OHD3, LDL-cholesterol, triglyceride, SBP, DBP, smoking history, drinking, hypertension, DM, renal dysfunction, cancer, vigorous work activity, annual household income, education, and race/ethnicity.

**Table 2:** Multivariate regression analyses of the association between obesity and AAC risk in NHANES.

	Age <52 years	Age >=52 years	Total
Non-adjusted			
** Non-** ** Obesity**	0	0	0
** Obesity**	0.45 (−0.06, 0.95) 0.09	−0.92 (−1.55, −0.28) <0.01	−0.65 (−1.18, −0.12) 0.02
Adjust I			
** Non-** **Obesity**	0	0	0
** Obesity**	0.40 (−0.16, 0.97) 0.17	−0.87 (−1.55, −0.20) 0.01	−0.63 (−1.20, −0.07) 0.03
Adjust II			
** Non-** **Obesity**	0	0	0
** Obesity**	0.30 (−0.36, 0.86) 0.42	−1.11 (−1.78, −0.25) <0.01	−0.78 (−1.36, −0.08) 0.02

Data are presented as β (95% CI) unless indicated otherwise.

Outcome: AAC scores.

Exposure: obesity or non-obesity

Non-adjusted model adjusts for: none.

Adjust I model adjust for: gender, smoking history, drinking, vigorous work activity, annual household income, education, and race/ethnicity.

Adjust II model adjust for: age, gender, 25OHD3, LDL-cholesterol, triglyceride, SBP, DBP, smoking history, drinking, hypertension, DM, renal dysfunction, cancer, vigorous work activity, annual household income, education, and race/ethnicity. Abbreviations: AAC, abdominal aortic calcification; SBP, systolic blood pressure; DBP, diastolic blood pressure; 25OHD3, 25-hydroxyvitamin D3; DM, diabetes mellitus.

Finally, we further analyzed the relationship between obesity and AAC score by stratification method (**Table 3**). Overall, the results were statistically significant in the age group ≥52 years, suggesting a negative correlation between obesity and AAC scores. In particular, the risk of AAC was lower for patients with obesity with the following characteristics: male, low LDL, low triglyceride, DM, non-cancer patient, smoking, drinking, vigorous work activity, low annual household income, education of 9 – 11th grades and non-Hispanic white.

**Table 3:** Association of obesity and AAC score in strata defined by sample characteristics.

	Age <52 years	Age >=52 years
Gender		
** Male**	0.60 (-0.07, 1.26) 0.08	-1.60 (-2.52, -0.68) 0.001
** Female**	0.35 (-0.53, 1.22) 0.44	-0.65 (-1.61, 0.31) 0.19
25OHD3, nmol/L		
** Low**	0.77 (0.01, 1.53) 0.05	-1.12 (-2.09, -0.15) 0.02
** High**	-0.06 (-0.76, 0.64) 0.87	-0.94 (-1.89, 0.00) 0.05
LDL-cholesterol, mmol/L		
** Low**	0.99 (0.10, 1.89) 0.03	-1.50 (-2.42, -0.58) 0.002
** High**	0.12 (-0.55, 0.79) 0.73	-0.51 (-1.45, 0.44) 0.29
Triglyceride, mmol/L		
** Low**	0.54 (-0.16, 1.24) 0.13	-1.41 (-2.47, -0.35) 0.01
** High**	0.31 (-0.66, 1.29) 0.53	-0.91 (-1.81, -0.01) 0.05
Hypertension		
** Yes**	0.06 (-1.08, 1.21) 0.92	-1.22 (-2.07, -0.37) 0.005
** No**	0.63 (0.02, 1.24) 0.04	-1.32 (-2.30, -0.34) 0.01
DM		
** Yes**	1.30 (-0.70, 3.31) 0.22	-2.29 (-3.56, -1.01) 0.0005
** No**	0.27 (-0.27, 0.81) 0.33	-0.85 (-1.62, -0.07) 0.03
Renal dysfunction		
** Yes**	-^#^	
** No**	0.47 (-0.07, 1.02) 0.09	-1.85 (-4.69, 0.98) 0.21
**Cancer**		-1.04 (-1.73, -0.36) 0.003
** Yes**	1.93 (-0.46, 4.32) 0.15	-0.24 (-1.70, 1.21) 0.74
** No**	0.39 (-0.17, 0.94) 0.18	-1.27 (-2.01, -0.52) 0.0009
Smoking (at least 100 cigarettes in life)		
** Yes**	0.34 (-0.34, 1.02) 0.33	-1.40 (-2.30, -0.51) 0.002
** No**	0.57 (-0.28, 1.43) 0.19	-0.61 (-1.59, 0.37) 0.22
Drinking (at least 12 alcohol drinks/1 year)		
** Yes**	0.71 (0.06, 1.36) 0.03	-1.10 (-1.89, -0.31) 0.006
** No**	-0.02 (-1.30, 1.25) 0.97	-0.79 (-2.08, 0.50) 0.23
Vigorous work activity		
** Yes**	0.06 (-1.08, 1.21) 0.92	-1.34 (-2.66, -0.03) 0.05
** No**	0.63 (0.02, 1.24) 0.04	-0.92 (-1.69, -0.15) 0.02
Annual household income		
** Low**	-0.57 (-1.54, 0.40) 0.26	-1.33 (-2.29, -0.36) 0.007
** Medium**	1.28 (0.39, 2.17) 0.01	-1.10 (-2.29, 0.08) 0.07
** High**	0.92 (-0.13, 1.97) 0.09	-0.41 (-2.11, 1.30) 0.64
Education		
** Less than 9th grade**	1.00 (-1.64, 3.64) 0.47	-1.14 (-3.35, 1.07) 0.32
** 9–11th** grades	1.05 (-0.67, 2.76) 0.24	-1.87 (-3.59, -0.15) 0.04
** High school graduate**	0.06 (-0.99, 1.11) 0.91	-0.53 (-1.93, 0.88) 0.46
** Some college or AA degree**	0.52 (-0.61, 1.66) 0.37	-1.02 (-2.17, 0.13) 0.08
** College graduate or above**	0.17 (-0.69, 1.04) 0.70	-1.22 (-2.76, 0.31) 0.12
Race/ethnicity		
** Mexican American**	0.72 (-1.18, 2.61) 0.46	-0.06 (-1.87, 1.74) 0.94
** Other Hispanic**	0.33 (-1.70, 2.36) 0.75	-1.78 (-4.06, 0.50) 0.13
** Non-Hispanic white**	0.40 (-0.24, 1.04) 0.23	-1.11 (-2.04, -0.18) 0.02
** Non-Hispanic black**	0.36 (-1.37, 2.08) 0.69	-1.06 (-2.58, 0.46) 0.17
** Other Race - Including ** **Multi-Racial**	-0.47 (-2.26, 1.33) 0.62	-1.73 (-3.88, 0.42) 0.12

Data are presented as β (95% CI) unless indicated otherwise.

Outcome: AAC scores.

Exposure: obesity or non-obesity.

AAC, abdominal aortic calcification; SBP, systolic blood pressure; DBP, diastolic blood pressure; 25OHD3, 25-hydroxyvitamin D3; DM, diabetes mellitus. Mean +/− SD for: age, AAC scores, 25OHD3, LDL-cholesterol, triglyceride, SBP and DBP. Percentage for: gender, smoking history, drinking, hypertension, DM, renal dysfunction, cancer, vigorous work activity, annual household income, education and race/ethnicity. # means less data, not calculated.

## Discussion

In our study, we found AAC became more common after the age of 50 years old. The average age of AAC individuals in our study was 59.3 years. The AAC scores were increased with age which was consistent to other reports as an age-related disease (23). The prevalence of AAC increased to 100% in both males and females when they were over 75 years of age (2).

As far as we know, this is the first report showing that obesity was negative association with the AAC. This conclusion is contrary to the finding of recent reports showing that obesity was associated with higher risk of AAC (24–27). After comparison, it was found that the reason for the difference between our study and other studies was due to the different ways of evaluating obesity. We define obesity using BMI, other studies have used weight-adjusted waist index (WWI) and a body shape index (ABSI). WWI and ABSI are a newly-developed parameter of obesity that more accurately estimates whole-body fat percentage (28, 29). The mechanism underlying the positive association between WWI/ABSI and AAC may be correlated with metabolic abnormalities. It is also possible that WWI/ABSI and AAC are a concomitant relationship related to age, and there is no inherent correlation (30).

Controversies about the obesity paradox have a long history (31). It has become increasingly apparent during the past half century that a relationship exists between obesity and CVD (32). The obesity paradox could be explained by the inherent limitations of both BMI and clinical studies (33). BMI does not differentiate between muscle mass and fat mass. Its assessment of body fat in older adults is not as accurate as that in younger adults (34). Perhaps it is precisely because of the difference in the accuracy of BMI in evaluating fat content between young and old people that the different results of this study appear. A limited number of previous studies have assessed the severity of CAC among those with obesity compared with those without obesity (14). Obesity was associated with increased progression of CAC in those at lower risk of CVD. But no baseline obesity measure was significantly associated with progression of CAC among those at higher risk for CVD. We found that the mean age of the group with high-risk of CVD was 57.9 years old, which is significantly higher than the mean age of the low-risk group (48.8 years old). Older age may be one reason why obesity is not positively associated with CAC progression in patients at high risk for CVD. Therefore, age stratification is necessary when analyzing the relationship between BMI and cardiovascular events. We did find an unusual relationship between BMI and AAC in relatively old age. Obese people aged 52 years and older were associated with a lower risk of AAC. This seems to provide new evidence for the obesity paradox.

How to explain the obesity paradox in the elderly? One finding show that obesity is often associated with increased survival time among people who have some serious injury or illness (35). In general, older people have a higher risk of disease than younger people. In this sense, it seems that our study as well as this one supports the obesity paradox. Beyond that, the obesity paradox is not entirely devoid of internal mechanisms. Some studies have summarized the molecular mechanisms by which adipose tissue protects against cardiovascular disease, such as efficient fat storage and lipid droplet formation, high adipogenesis capacity, low extracellular matrix fibrosis, angiogenesis potential, adipocyte browning and low macrophages infiltration/activation (33). The obesity paradox is also attributable to increased cardiac lipid supply from adipose lipolysis in the fasting cycle due to systemic insulin resistance and adiposity (36). The specific molecular mechanism to explain obesity paradox in old age needs to be confirmed by basic research in the future.

In summary, obesity was associated with decreased AAC risk for adults aged 52 years or older in US. In the future, specific molecular mechanism and larger population-based studies stratified by age exploring the relationship between obesity and AAC may be warranted.

### Limitations

Despite the strengths of this study, several limitations should also be considered. First, we examined a cross-sectional population sample in 2013–2014 from NHANES, which was the only investigation of the AAC that NHANES has done so far. Second, these results are based on U.S. adults with obesity, which may limit the generalizability to other populations. Finally, as with any observational study, we cannot exclude the possibility of residual or unmeasured confounding effects.

## Data availability statement

The original contributions presented in the study are included in the article/supplementary materials. Further inquiries can be directed to the corresponding author.

## Ethics statement

The survey protocol was approved by NCHS Ethics Review Board (https://www.cdc.gov/nchs/nhanes/irba98.htm), and all participants have written informed consent. The studies were conducted in accordance with the local legislation and institutional requirements. The participants provided their written informed consent to participate in this study. Written informed consent was obtained from the individual(s) for the publication of any potentially identifiable images or data included in this article.

## Author contributions

TG: Conceptualization, Data curation, Formal analysis, Funding acquisition, Investigation, Methodology, Project administration, Resources, Software, Supervision, Validation, Visualization, Writing – original draft, Writing – review & editing. BC: Conceptualization, Supervision, Visualization, Writing – review & editing. LH: Data curation, Resources, Visualization, Writing – original draft, Writing – review & editing. ZL: Data curation, Investigation, Methodology, Validation, Writing – review & editing. HZ: Formal analysis, Methodology, Writing – review & editing. SS: Data curation, Investigation, Methodology, Software, Writing – review & editing.
